# Heterochrony and repurposing in the evolution of gymnosperm seed dispersal units

**DOI:** 10.1186/s13227-022-00191-8

**Published:** 2022-02-16

**Authors:** Juca A. B. San Martin, Raúl E. Pozner, Verónica S. Di Stilio

**Affiliations:** 1 Instituto de Botánica Darwinion (IBODA, CONICET & ANCEFN), Labardén 200, C.C. 22, B1642HYD San Isidro, Buenos Aires Argentina; 2 Department of Biology, University of Washington, Seattle, WA 98195-1800 USA

**Keywords:** Bracts, Cellulosic fibers, Fruit-like, Heterochrony, Histochemistry, Gnetales, Gymnosperm, Mucilage, Ontogeny, Seed dispersal

## Abstract

**Background:**

Plant dispersal units, or diaspores, allow the colonization of new environments expanding geographic range and promoting gene flow. Two broad categories of diaspores found in seed plants are dry and fleshy, associated with abiotic and biotic dispersal agents, respectively. Anatomy and developmental genetics of fleshy angiosperm fruits is advanced in contrast to the knowledge gap for analogous fleshy structures in gymnosperm diaspores. Improved understanding of the structural basis of modified accessory organs that aid in seed dispersal will enable future work on the underlying genetics, contributing to hypotheses on the origin of angiosperm fruits. To generate a structural framework for the development and evolution of gymnosperm fleshy diaspores, we studied the anatomy and histochemistry of *Ephedra* (Gnetales) seed cone bracts, the modified leaves surrounding the reproductive organs. We took an ontogenetic approach, comparing and contrasting the anatomy and histology of fleshy and papery-winged seed cone bracts, and their respective pollen cone bracts and leaves in four species from the South American clade.

**Results:**

Seed bract fleshiness in *Ephedra* derives from mucilage accumulated in chlorenchyma cells, also found in the reduced young leaves before they reach their mature, dry stage. Cellulosic fibers, an infrequent cell type in gymnosperms, were found in *Ephedra*, where they presumably function as a source of supplementary apoplastic water in fleshy seed cone bracts. Papery-winged bract development more closely resembles that of leaves, with chlorenchyma mucilage cells turning into tanniniferous cells early on, and hyaline margins further extending into “wings”.

**Conclusions:**

We propose an evolutionary developmental model whereby fleshy and papery-winged bracts develop from an early-stage anatomy shared with leaves that differs at the pollination stage. The ancestral fleshy bract state may represent a novel differentiation program built upon young leaf anatomy, while the derived dry, papery-winged state is likely built upon an existing differentiation pattern found in mature vegetative leaves. This model for the evolution of cone bract morphology in South American *Ephedra* hence involves a novel differentiation program repurposed from leaves combined with changes in the timing of leaf differentiation, or heterochrony, that can further be tested in other gymnosperms with fleshy diaspores.

**Supplementary Information:**

The online version contains supplementary material available at 10.1186/s13227-022-00191-8.

## Background

Plants are sessile organisms with limited opportunities for gene flow, mainly via spores (the haploid stage) or seeds (the diploid stage). Seeds represent a major innovation in the history of land plants that ultimately enabled long-distance dispersal of a dormant embryo [1] wrapped in nutritive tissue, via abiotic or biotic agents. Extant seed plants consist of two major clades: gymnosperms, with naked ovules, and angiosperms, with ovules contained in ovaries that develop into fruits after pollination and fertilization. Angiosperm fruits have evolved highly diverse morphologies, with two broad categories consisting of dry and fleshy. While gymnosperms do not have true fruits in the botanical sense, structures other than the angiosperm ovary can perform comparable functions in seed dispersal, similarly becoming fleshy or dry and winged [2].

Strong selective pressures for the dispersal of progeny away from the maternal plant have led to multiple adaptations in seed dispersal units, or diaspores [3]. On the one hand, high elevation environments that typically exhibit high wind speeds, low vegetation cover and low animal density favor adaptations to wind dispersal such as winged, pappose, or light diaspores. On the other hand, lower elevation environments with higher vegetation cover and animal density favor dispersal by animals, typically in the form of fleshy diaspores [4]. Fleshy structures that aid in animal seed dispersal have evolved repeatedly and independently from different organs in gymnosperms, either within the ovule or from subtending structures [5].

**Fig. 1:**
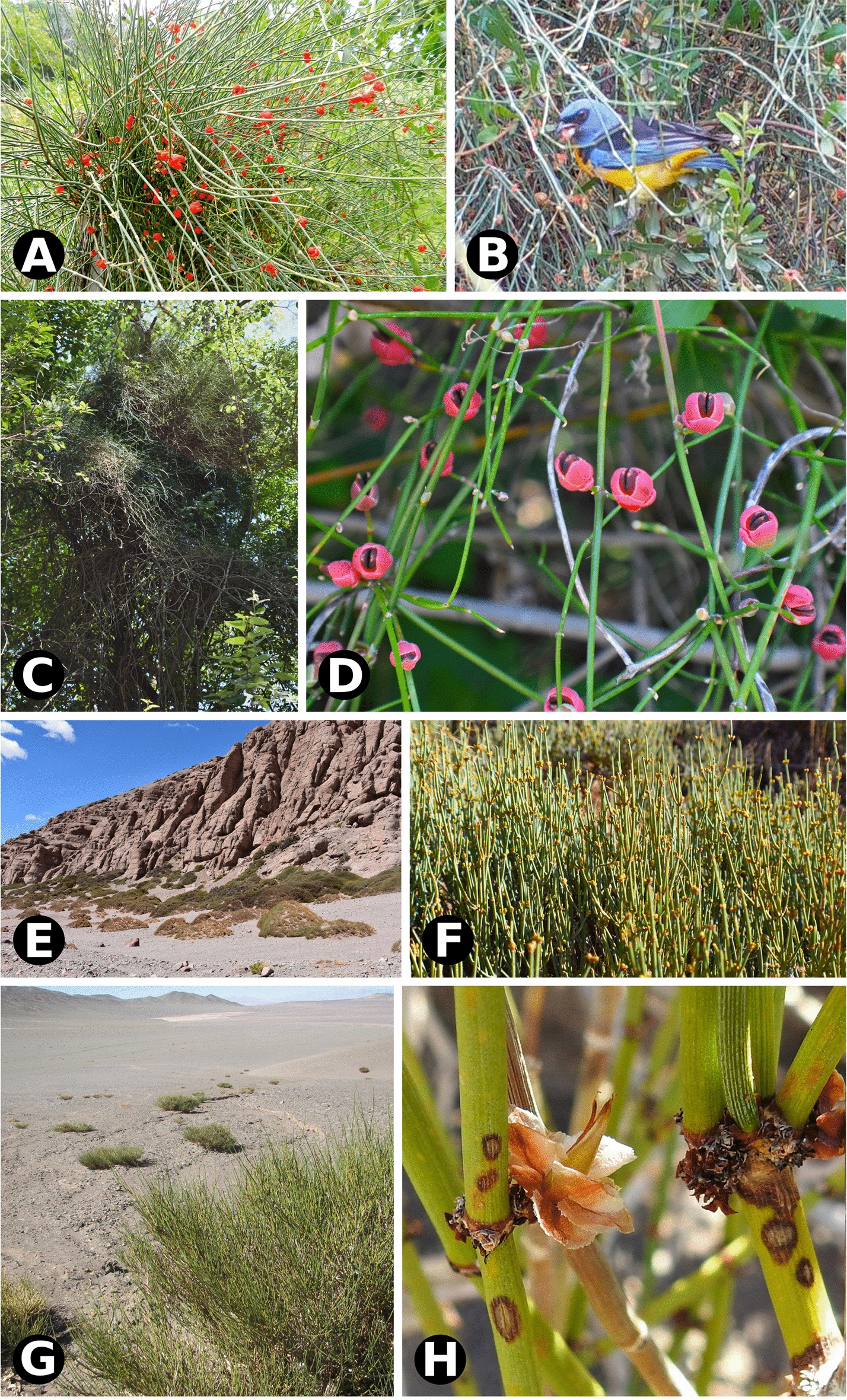
Natural environment, habit and dispersal biology of *Ephedra* species studied. **A** *E. triandra* with seed cones; **B** Bird disperser *Rauenia bonariensis*, “naranjero” feeding on the fleshy cones (Anillaco, la Rioja); photos in **A**, **B** by Adriana Aranda-Rickert. **C** *E. tweediana* climbing on a tree; **D** detail of fleshy cones (El Rodeo, Catamarca). **E** *E. breana* in high elevation semi-arid environment; **F** plant detail (Laguna Brava, La Rioja). **G** Sparse populations of *E. multiflora* in high elevation semi-dessert environment; photo by Javier Torréns; **H** detail of plant habit (Laguna Brava, La Rioja); photo by Adriana Aranda-Rickert

While there has been substantial progress in understanding the anatomical, developmental and genetic basis of angiosperm fleshy fruits [6], much less is known about analogous fleshy structures of multiple origin in gymnosperms [7, 8], except for *Ginkgo* and certain Cycadales [9]. The idea that a basic genetic toolkit involving MADS box genes may be at play in all seed plant fleshy diaspores is appealing [10, 11]. However, addressing the potential co-option of genetic elements will require more in-depth knowledge of developmental morphology and anatomy in fleshy structures with distinct ontogenetic origin across gymnosperms. *Ephedra* (Gnetales) is an ideal system to investigate the evolution and development of fruit-like function in gymnosperms, since it includes species with fleshy and non-fleshy diaspores dispersed by animals or wind, respectively [12] (Fig. 1). A central argument for proposing to develop *Ephedra* into an evo-devo model lineage is indeed its contribution to the study of convergent fruit‐like function in seed plants, by focusing on the fleshy character in non-angiosperm seed dispersal [13]. Within Gnetales, fleshy seed cone bracts are a distinctive ancestral feature found in *Ephedra,* where non-fleshy, papery-winged or coriaceous seed cone bracts have arisen independently multiple times [14]**.**

The *Ephedra* dispersal unit is the seed cone, found in female individuals of this dioecious gymnosperm, consisting of three pairs of decussate bracts and two ovules (except for a few species with three ovules and verticillate bracts). *Ephedra* seed cone bract anatomy and histology is relatively simple at the pollination stage (when the female gametophyte is fully mature, with a pollination drop), typically consisting of a tanniniferous epidermis, a parenchymatous mesophyll (with or without fibers and tanniniferous cells), and two parallel vascular bundles surrounded by transfusion tissue [15, 16]. This early pattern was presumed equivalent between fleshy and non-fleshy bracts, except for the number of mesophyll layers and the width of the membranous margins. However, upon closer scrutiny of the published descriptions, we found that fleshy seed cone bracts can actually develop from different anatomies at the pollination stage: from parenchymatous mesophyll with or without tannins (e.g., *Ephedra equisetina*, *E. foeminea*, *E. minuta*, *E. saxatilis*) or from parenchymatous mesophyll with scattered (e.g. *E. fragilis*, *E. aphylla*, *E. altissima*) or subepidermal fibers (e.g., *E. tweediana*, *E. distachia*, *E. sarcocarpa*). This novel insight prompted us to conduct more detailed comparative work in order to clarify the developmental and structural basis of fleshy *Ephedra* bracts, and to contribute towards a better understanding of this important innovation. To that end, we included in our study the anatomy of putatively homologous pollen cone bracts (found in male individuals) and leaves, in order to gain a full evolutionary perspective.

Here, we investigate how seed accessory structures become fleshy at the anatomical and histological level in the gymnosperm lineage *Ephedra*. To that end, we ask the following questions: (a) What is the developmental anatomy of fleshy seed cone bracts, and how does it differ from that of papery-winged bracts, and non-fleshy pollen cone bracts? (b) Are there new cell or tissue types associated with the development of fleshiness? (c) Considering that cone bracts are modified leaves and hence homologous, do fleshy bracts share anatomical elements with leaves that suggest repurposing? To address these questions, we studied seed cone bract development in four species of *Ephedra* belonging to the South American clade, integrating morphology, anatomy, and histochemistry in an ontogenetic framework. Fleshy seed cone bracts of *Ephedra breana*, and sister species *E. triandra* and *E. tweediana* were compared to their respective non-fleshy pollen cone bracts and vegetative leaves, and to papery-winged seed cone bracts of *E. multiflora*. We end by summarizing our observations into a structural working model and by proposing hypotheses on the evolution of fleshy bracts from leaves in this intriguing gymnosperm lineage. The investigation of developmental patterns in fleshy and non-fleshy *Ephedra* diaspores uncovered more general processes such as anatomical repurposing and changes in developmental timing that help explain the emergence of this innovation.

## Results

### Developmental staging, comparative anatomy, and histology of seed cone bracts

**Fig. 2:**
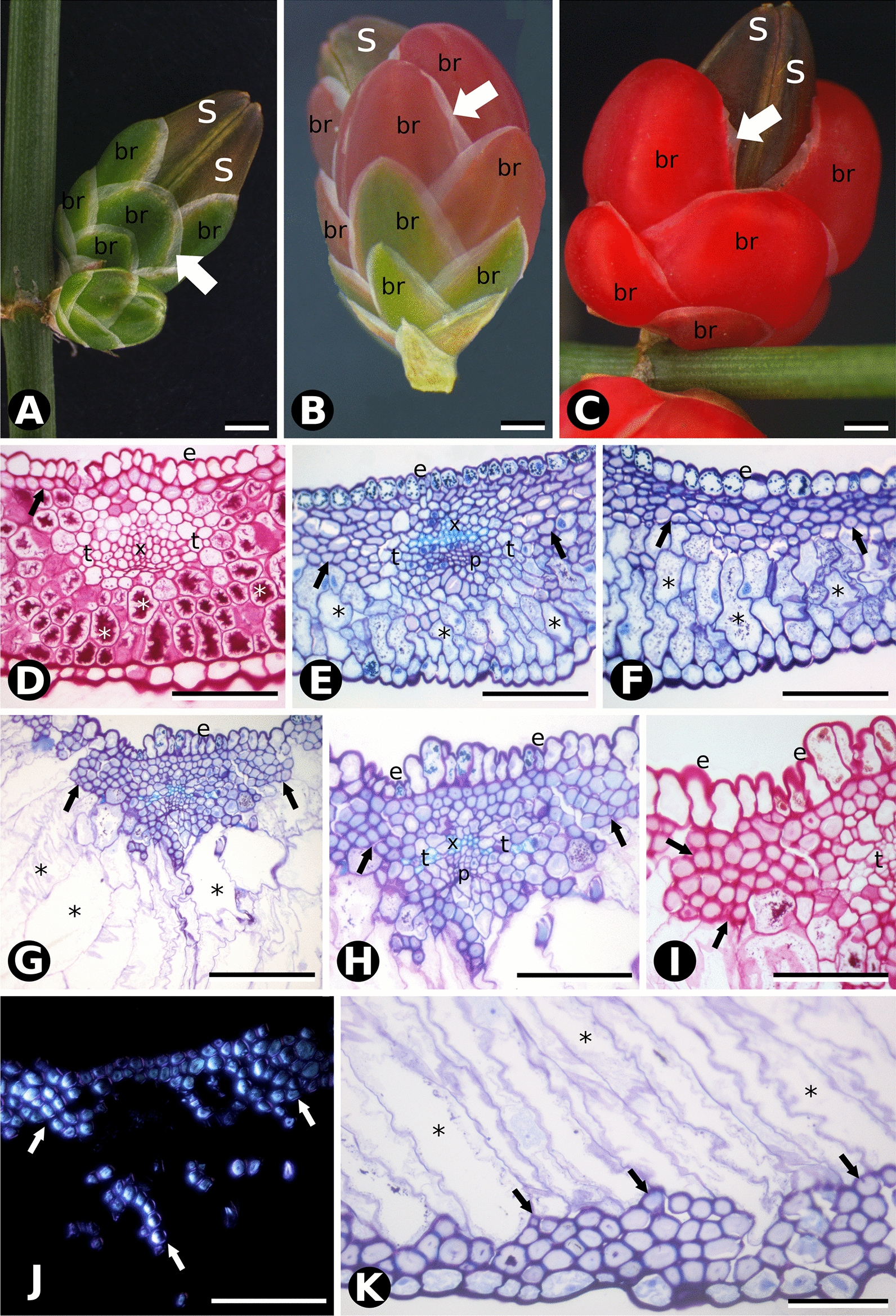
Comparative morphology and bract anatomy of *Ephedra triandra* seed cones at three developmental stages: **A** green (Stage 1), **B** red (Stage 2), and **C** fleshy (Stage 3). The white arrow shows a papyraceous lateral region that decreases as bracts (br) and seeds (s) mature. **D** Cross section of a green bract stained with PAS showing a continuous single layer of cellulosic fibers beneath the epidermis (black arrow), and a mesophyll of cells with substantial amounts of insoluble carbohydrates (*). Adaxial epidermis (e), xylem (x) and transfusion tissue (t). **E**, **F** Cross sections of red bracts stained with PAS and toluidine blue O, showing the proliferation of cellulosic fibers (black arrows) beneath the adaxial epidermis. Mesophyll cells (*) no longer contain insoluble carbohydrates. **G** Cross section of red and fleshy bracts showing an adaxial epidermis with columnar cells and cellulosic fibers organized in several subepidermal layers (arrows). Mesophyll cells (*) look enlarged, with thin primary walls and enlarged vacuoles. **H** Detail of the vascular bundle in **G**, adaxial epidermis, xylem and transfusion tissue. **I** Detail of cellulosic fibers (arrows) showing intense PAS staining on their primary walls (black arrows) and lighter staining on their secondary walls. Adaxial epidermis and transfusion tissue. **J** Polarized light microscopy showing the intense birefringence of cellulosic fibers in cross section (arrows). **K** Detail of the abaxial face showing the distribution of cellulosic fibers (arrows) in subepidermal layers. Mesophyll cells (*) are enlarged, with thin, sinuous walls and enlarged vacuoles in their cytoplasm. Scale bars: 1 mm (**A**–**C**); 100 µm (**D**); 50 µm (**E**, **F**, **I**–**K**); 100 µm (**G**, **H**)

**Fig. 3:**
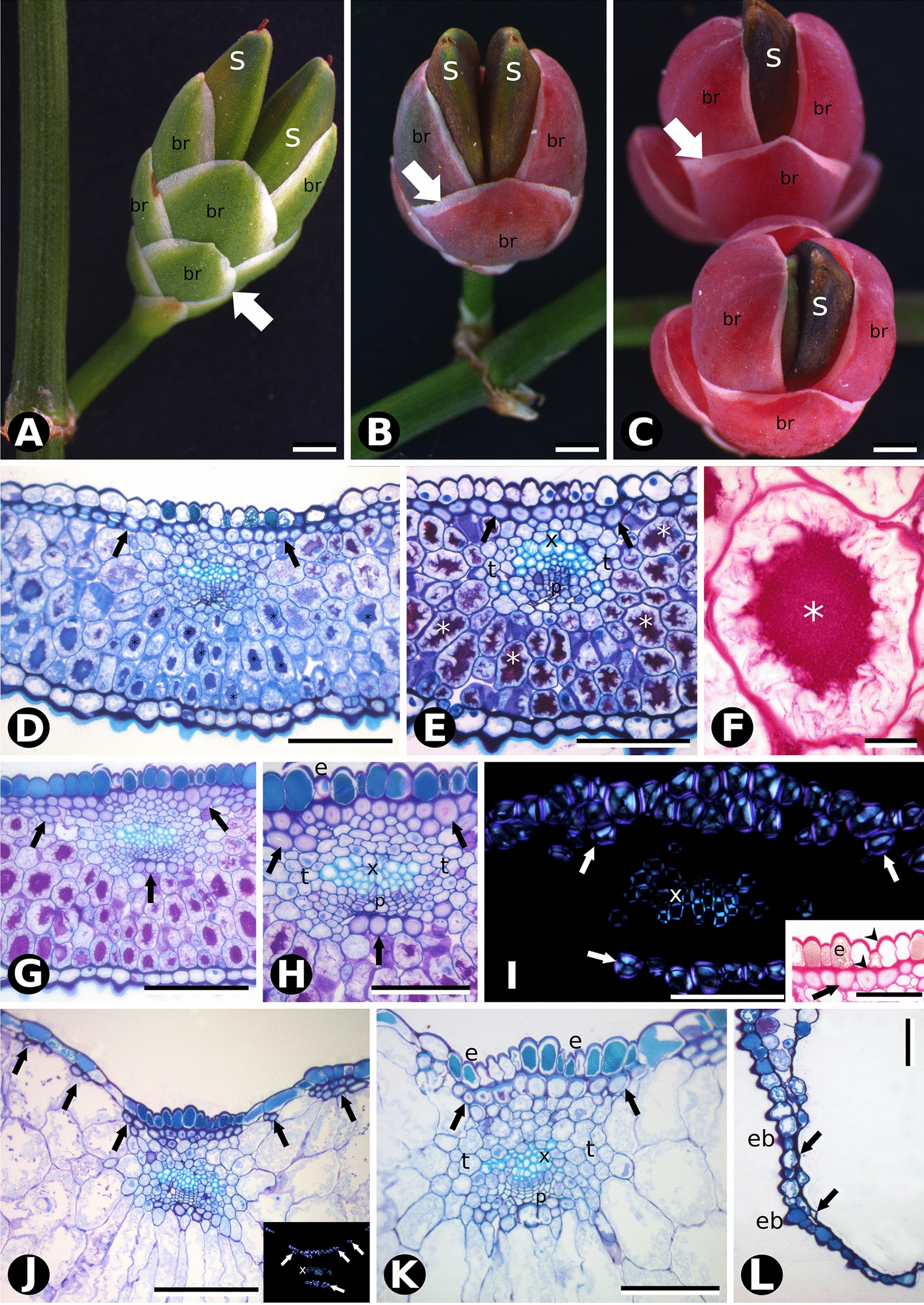
Comparative morphology and bract anatomy of *Ephedra tweediana* seed cones at three developmental stages: **A** green (Stage 1); **B** red (Stage 2), and **C** fleshy (Stage 3). White arrows show the papyraceous lateral region of the bracts (br); two seeds (s) are present per cone. **D** Cross section of a green bract stained with toluidine blue O and PAS showing a homogeneous mesophyll consisting of cells with substantial amounts of insoluble carbohydrates (*) and cellulosic fibers (black arrows) distributed in a single adaxial subepidermal layer. **E** Detail of vascular bundle in **D**, with mesophyll cells with substantial amounts of insoluble carbohydrates, phloem (p), xylem (x) and transfusion tissue (t). **F** Detail of mesophyll cell stained with PAS showing its vacuole filled with insoluble carbohydrates. **G** Overview of a red bract in cross section showing an increase in the number of cellulosic fiber layers (black arrows). **H** Detail of the vascular bundle shown in **G**, with adaxial epidermal cells (e) filled with phenolic compounds (tannins), and cellulosic fibers organized into one or two subepidermal layers (black arrows). **I** Polarized light microscopy showing the intense birefringence of cellulosic fibers in cross section (white arrows). Inset, detail of cellulosic fibers showing intense PAS staining on primary walls (arrowheads) and weak staining on secondary walls (black arrow). Adaxial epidermis (e) with thickening of the external and internal periclinal walls (arrowheads). **J**–**L** Cross section of fleshy bracts (Stage 3). **J** Cellulosic fibers (black arrows) are organized in separate bundles of one or two layers beneath the adaxial epidermis. Mesophyll cells are large with thin, sinuous walls and devoid of cytoplasmic content. Inset: polarized light microscopy showing the intense birefringence of cellulosic fibers (white arrows) and xylem. **K** Detail of the vascular bundle area beneath the adaxial epidermis with cellulosic fibers (black arrows) above, phloem, xylem and transfusion tissue. **L** Detail of papery lateral region of the bract, consisting solely of epidermis, with adaxial cells collapsed and reduced to the periclinal, juxtaposed walls (black arrows) and abaxial epidermis (eb) with tanniniferous cells. Scale bars: 1 mm (**A**–**C**); 100 µm (**D**, **E**, **G**, **J**–**L**); 50 µm (**I**); 20 µm (**F**)

Fleshy seed cone bracts differ in general morphology and anatomy from papery-winged bracts since pollination. At Stage 1 (non-fleshy green, pollination stage) *Ephedra triandra* (Fig. 2) and *E. tweediana* (Fig. 3) seed cone bracts are wide triangular to wide ovate, with thin, hyaline margins and a thick, green central region running longitudinally (Figs. 2A and 3A). The adaxial (inner) epidermis had a similar structure to the abaxial (outer) epidermis, except that the latter had a thicker cuticle layer on the outer tangential walls (Figs. 2D and 3D, E). Sunken stomata are present on the abaxial epidermis only, while tanniniferous cells are found on both sides (Fig. 3D). Two longitudinal vascular bundles run along each bract, surrounded by transfusion tissue (Figs. 2D and 3D, E). The mesophyll consists of 4–5 layers of mucilage chlorenchyma abaxially (Figs. 2D and 3D, F) and one adaxial layer of cellulosic fibers, with vascular bundles running along the boundary (Figs. 2D and 3D, E). Mucilage chlorenchyma contains cells with intense PAS and Alcian Blue staining in the central vacuole (Fig. 3F, Table 1 and Additional file 1). Bracts have 8- to 15-cell-wide hyaline margins without mesophyll, consisting solely of an epidermis with adaxial cells that are mostly collapsed and reduced to their juxtaposed tangential walls (Fig. 3L).

**Table 1:** Summary of histochemical and microscopy tests on pollen and seed cone bracts

Histochemical test	Pollen cone bract				Seed cone bract				
	Epidermis	Fibers		Parenchyma	Epidermis (Stage 1–3)	Fibers (Stage 1–3)		Parenchyma	
		Primary wall	Secondary wall			Primary wall	Secondary wall	(Stage 1–2)	(Stage 3)
Toluidine blue O	Green	Blue	Not stained	Blue	Green	Blue	Not stained	Blue	Blue
PAS	−−−	+++	−−−	+++	−−−	+++	−−−	+++	−−−
Alcian blue	−−−	−−−	−−−	+++	−−−	−−−	−−−	+++	−−−
Ruthenium Red	−−−	+++	−−−	−−−	−−−	+++	−−−	−−−	−−−
IKI (Lugol)	−−−	−−−	−−−	−−−	−−−	−−−	−−−	−−−	−−−
Polarized light	−−−	+++	+++	−−−	−−−	+++	+++	−−−	−−−
Coomassie blue R-2503	−−−	++−	−−−	+++*	−−−	++−	−−−	+++*	−−−
Sudan Black B	−−−	−−−	−−−	−−−	−−−	−−−	−−−	−−−	−−−

Cytochemical test specificity: toluidine blue O—polyanionic acid groups: blue, phenolic compounds: green, pectins: purple [46]; PAS—insoluble polysaccharides [44]; Alcian blue—mucilage [42, 45]; ruthenium red—pectins and IKI (Lugol)—starch [47]; polarized light—birefringent crystalline fibers [48]; Sudan Black B—lipids (including phospholipids) [49]. (+++) strong, (++−) positive, (+−) weak, (−−) none observed, (*) peripheral to vacuole

From developmental stages 1 to 2, mucilage chlorenchyma cells became enlarged in both species (Figs. 2E, F and 3G, H). In *E. triandra*, several additional layers of cellulosic fibers developed under the epidermis on both sides (Fig. 2E, F, K). In *E. tweediana*, a second layer of subepidermal cellulosic fibers developed adaxially (Fig. 3G–I). Vascular bundles were found closer to the adaxial side of the bract, resulting in xylem and transfusion tissue being in direct contact with the cellulosic fibers (Figs. 2E, F and 3G–I).

Seed cone bracts appeared to grow by expanding their green area at the expense of the hyaline margin, turning first red (Figs. 2B, E, F and 3B, G–I), and then fleshy (Figs. 2C, G–K and 3C, J, K). Throughout differentiation, mesophyll cells divided, expanded, and developed a large central vacuole, their mucilage content no longer identifiable with PAS (Figs. 2E–G, K and 3J, K) nor Alcian blue (Table 1, Additional file 1). This process involved both cell division and expansion (Figs. 2G, K and 3J, K), since the number of mesophyll cell layers increased from 8–14 to up to 30. Subepidermal cellulosic fibers persisted as a continuous multilayer throughout bract differentiation in *E. triandra* (Fig. 2G–J). In *E. tweediana*, the subepidermal layer of cellulosic fibers did not follow bract expansion, separating instead into isolated bundles (Fig. 3J, K).

**Fig. 4:**
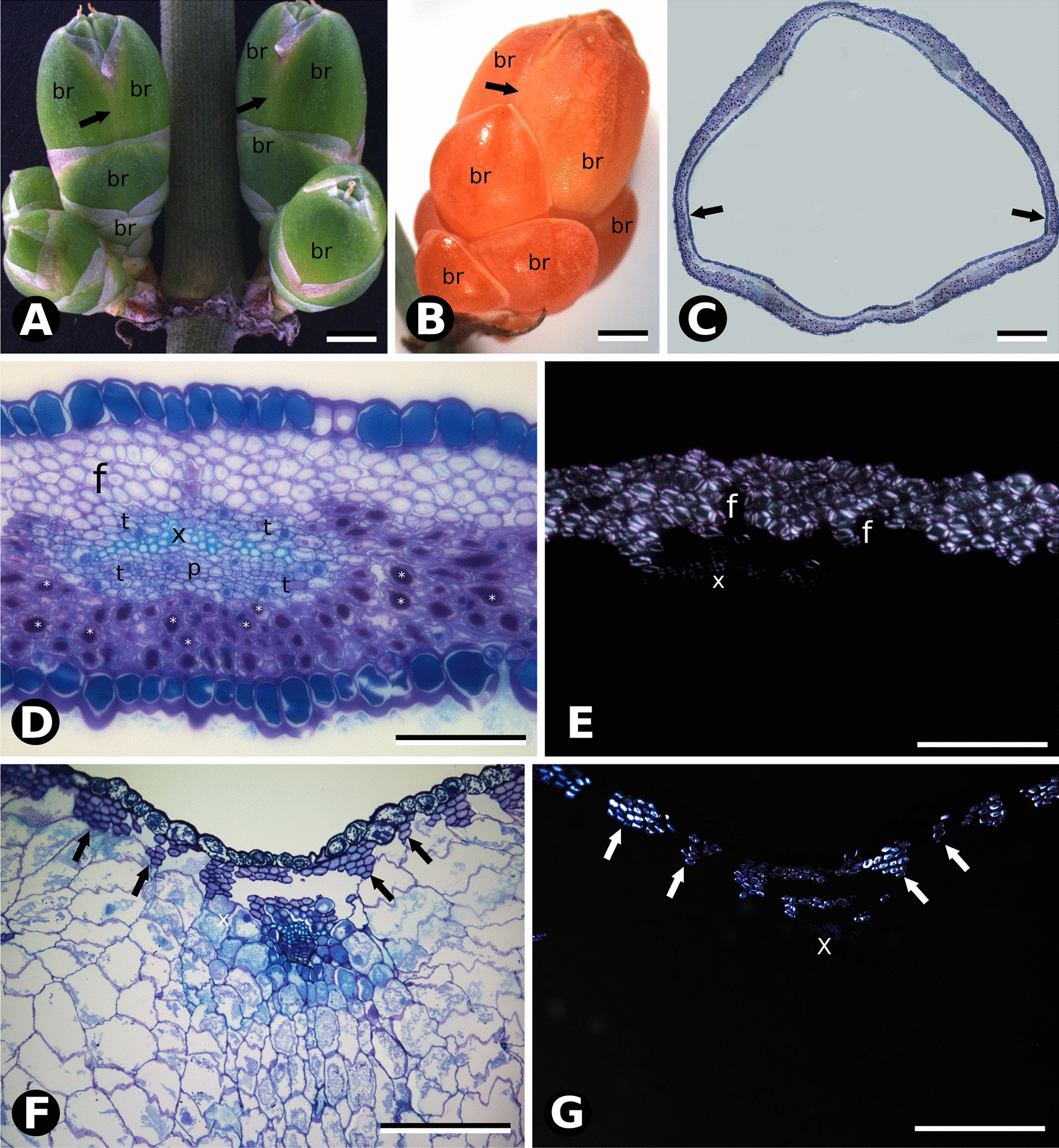
Comparative morphology and bract anatomy of *E. breana* seed cones. **A** Seed cone in Stage 1, with green bracts (br). Black arrows indicate the fused region of the distal bracts. **B** Seed cone at Stage 3, with fleshy red bracts. Black arrows indicate the fused region of the distal bracts. Note the highly developed distal bracts that completely enclose the seeds, which are not visible as in the other species studied. **C** Overview of anatomical section in the region of seed cone bract fusion (black arrows). **D** Cross section of seed cone bract at Stage 1 stained with toluidine blue O and PAS showing large tannin content in cells of the adaxial epidermis. Cellulose fibers (f) are grouped into layers on the adaxial side. A vascular bundle is surrounded by transfusion tissue (t), phloem (p) and xylem (x). Mucilaginous parenchyma is evident abaxially, and the abaxial epidermis is composed of cells with tannins and stomata. **E** Polarized light microscopy of section in **D** showing the birefringence of cellulosic fibers (f) and xylem. **F** Cross section of the seed cone bract at Stage 3 stained with toluidine blue and PAS showing increased of cell size and loss of carbohydrates from the cytoplasm of mucilaginous parenchyma cells. Fiber layers (arrows) are reduced in number and grouped in bundles beneath the adaxial epidermis. **G** Polarized light microscopy of the section in **F** showing the birefringence of cellulosic fibers (white arrows) and xylem. Scale bars: 1 mm (**A**, **B**); 200 µm (**C**); 100 µm (**D**–**G**)

At developmental Stage 1, *E. breana* seed cone bracts became fused at the base (Fig. 4A, C), their mesophyll consisting of 2–6 adaxial layers of cellulosic fibers and 4–6 abaxial layers of mucilage chlorenchyma (Fig. 4D, E). As in the species with fleshy bracts, anatomical changes from developmental Stage 2 to 3, when bracts turn red and fleshy (Fig. 4B), also involved division and expansion of mucilage cells (Fig. 4F, G). Adaxial cellulosic fibers did not accompany bract expansion, resulting in their separation into several subepidermal bundles (Fig. 4F, G).

**Fig. 5:**
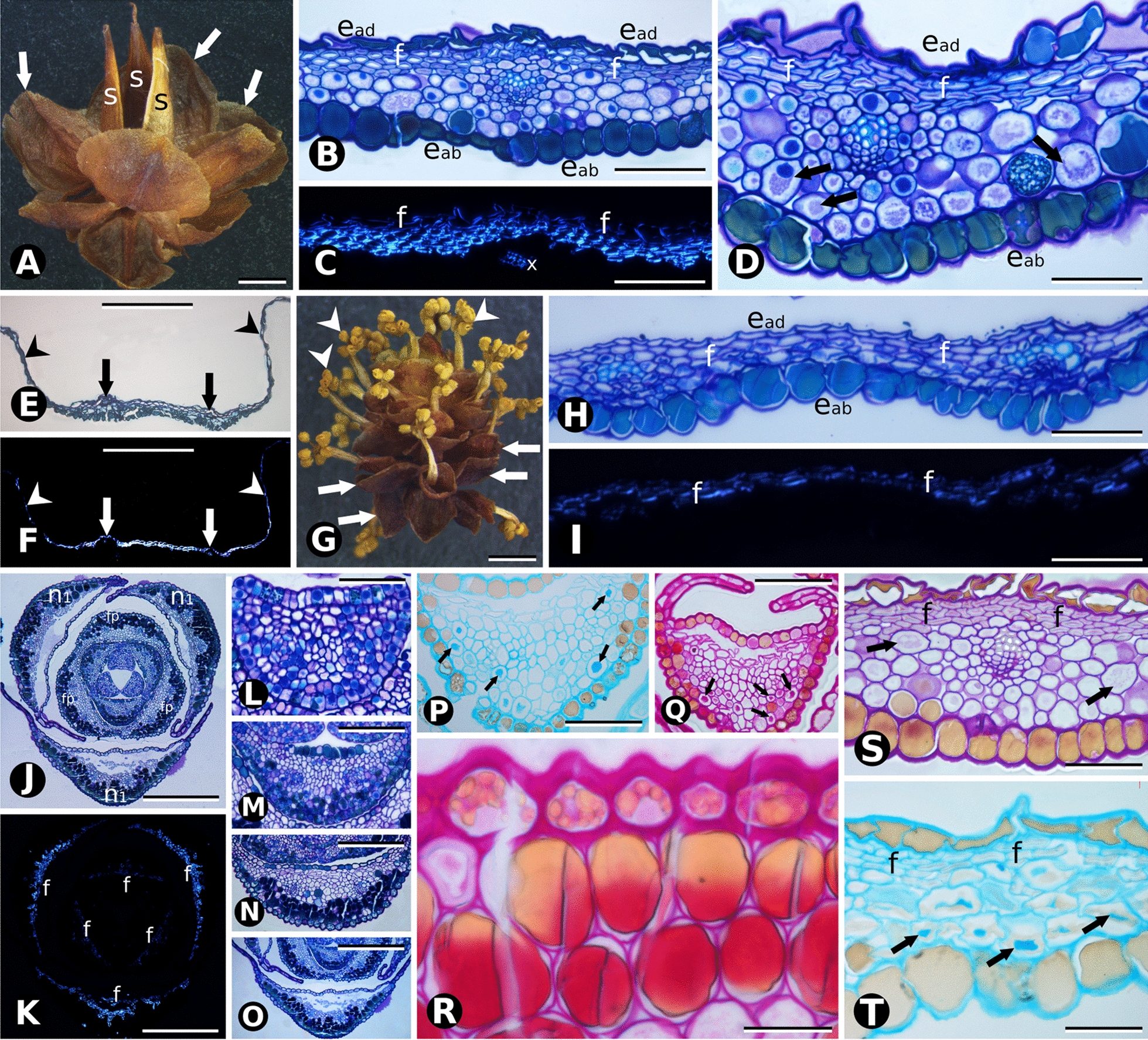
Morphology and anatomy of seed and pollen cone bracts and leaves of *E. multiflora*. **A** Seed cone with three seeds (s) and papery-winged bracts (arrow). **B**–**D** Cross section of the papery-winged seed cone bract (young green stage). **C** Polarized light microscopy of section in **B**. **D** Detail of vascular bundles and mucilage chlorenchyma cells (arrows). **E**, **F** General appearance of papery-winged seed cone bract in cross section, stained with PAS and toluidine blue O. Arrows indicate the two vascular bundles, and arrowheads the lateral papyraceous area. **G** Pollen cone after pollination, with open microsporangia (arrowheads) and papery-winged bracts (arrows). **H**, **I** Cross section of pollen cone bract. **I** Polarized light microscopy of section in **H**. **J**–**O** Leaf development. **J** Cross section of a terminal bud, with leaves from node 1 (n1) and leaf primordia (p). **K** Polarized light microscopy of section in **J**. **L**–**O** Detail from the youngest leaf primordia to a node 1 leaf. **P**, **Q** Histochemistry of a leaf primordium (same stage as **L**) revealing mucilage chlorenchyma cells (arrows), **P** Alcian Blue, **Q** PAS. **R** Mucilage cells turning into tanniniferous cells in a node 1 leaf. **S**, **T** Cross section of a seed cone bract with mucilage cells (arrows). **S** PAS. **T** Alcian Blue. Adaxial epidermis (e_ad_), abaxial epidermis (e_ab_), fibers (f). Scale bars: 1 mm (**A**, **G**); 500 µm (**J**, **K**); 400 µm (**E**, **F**, **O**); 300 µm (**N**); 200 µm (**B**, **C**, **H**, **I**, **M**); 100 µm (**D**, **L**, **P**, **Q**, **S**); 50 µm (**R**, **T**)

At an early green stage, *Ephedra multiflora* seed cone bracts had 3–5 abaxial layers of mucilage chlorenchyma in the mesophyll that progressively turned into tanniniferous cells (Fig. 5A–D) and became papery by Stage 1 (Fig. 5E, F). These bracts lacked mucilage chlorenchyma and the resulting gelatinous texture from Stage 1 onwards, instead developing extended, papery hyaline margins, or “wings” (Fig. 5E, F). While the epidermis had the anatomical features described above for the fleshy species, little remained of the mesophyll in the middle region (Fig. 5E, F). Both vascular bundles were still discernible and the mesophyll was reduced to two to three layers of subepidermal cellulosic fibers adaxially, and one to two abaxial tanniniferous layers (Fig. 5E, F). The hyaline bract margins were 55–60 cells wide without mesophyll, consisting solely of an epidermis of collapsed cells, reduced to juxtaposed tangential walls (Fig. 5E, F). Thus, papery-winged and fleshy seed cone bracts initially share similar anatomy at the early green stage, and later differ in the fate of the mucilage chlorenchyma, which turns either fleshy and red or dry and tanniniferous.

### Comparative anatomy and histology of pollen cone bracts

**Fig. 6:**
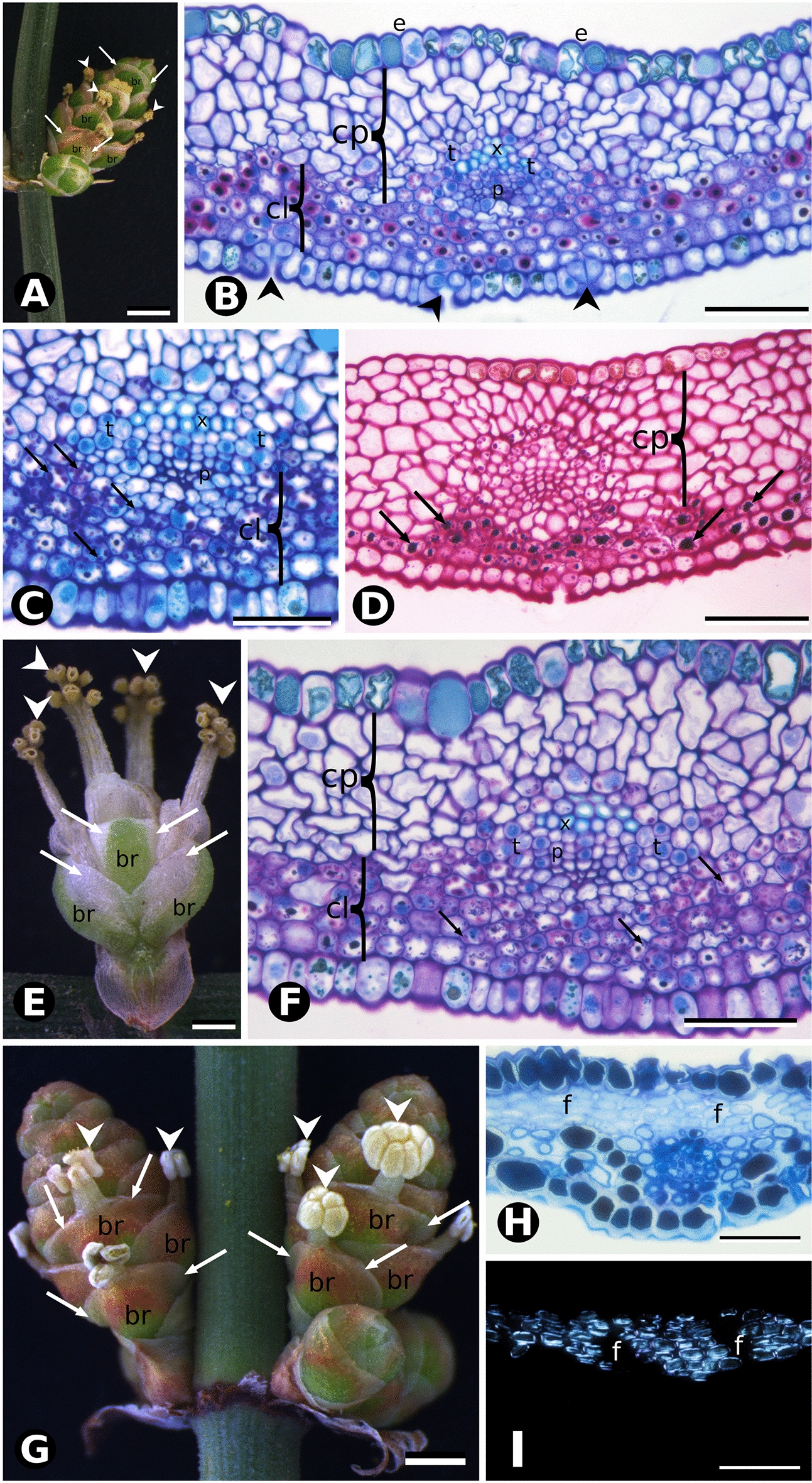
Comparative morphology and bract anatomy of *Ephedra* pollen cones.*Ephedra triandra* (**A**–**D**), *E. tweediana* (**E**, **F**) and *E. breana* (**G**–**I**). **A** Overall appearance of *E. triandra* pollen cone after pollination, green bracts (br) with hyaline margins (arrows) enclose one microsporangiophore each (arrowheads), with multiple microsporangia (pollen sacs) at their tips. **B** Cross section of *E. triandra* pollen cone bract stained with toluidine blue O and PAS. Adaxial epidermis (e) with tanniniferous cells with large phenolic content (tannin). Heterogeneous mesophyll with adaxial, compact parenchyma (cp) without intercellular spaces and abaxial chlorenchyma (cl) with substantial amounts of insoluble carbohydrates (mucilage) inside the vacuoles, evidenced by strong PAS staining (magenta to purple). Vascular bundle with poorly developed phloem (p), xylem (x) and transfusion tissue (t) at the interface between parenchyma types. Stomata (arrowheads) on the abaxial epidermis. **C** Vascular bundle in cross section with toluidine blue O. Chlorenchyma cells containing multiple chloroplasts (arrows). **D** Same bract stained with PAS showing substantial amounts of insoluble carbohydrates (arrows) within chlorenchyma cells. **E** Pollen cone of *E*. *tweediana* after pollination. Green bracts with white hyaline margins (arrows) enclose one microsporangiophore each (arrowheads), with multiple microsporangia (pollen sacs) at their tips. **F** Cross section of pollen cone bract with toluidine blue O showing the same anatomical pattern as in *E. triandra*. **G** Pollen cone of *E. breana* at anthesis. Microsporangia (arrowheads) at anthesis (left cone) and pre-anthesis (right cone), hyaline margins (arrows) on bracts. **H** Cross section of pollen cone bract at anthesis stained with toluidine blue O and PAS. Mesophyll with cellulosic fibers (f) and tanniniferous cells, without insoluble carbohydrates (mucilage). **I** Polarized microscopy of bract section in **H** showing birefringence of cellulosic fibers (f). Scale bars: 1 mm (**A**, **E**, **G**); 50 µm (**C**, **H**, **I**); 100 µm (**B**, **D**, **F**).

Pollen cone bracts of fleshy species also differ in general morphology and anatomy from those of the papery species since the pollination stage (Stage 1). Pollen cone bracts of the fleshy species *Ephedra triandra* (Fig. 6A–D) and *E. tweediana* (Fig. 6E, F) had the same external morphology and internal anatomy. They are also similar to the seed cone bracts at Stage 1, in their epidermis, abaxial stomata, vasculature, and margins (Fig. 6B–D, F). However, their mesophyll consists of 4–5 layers of compact parenchyma adaxially and 4–5 layers of mucilage chlorenchyma abaxially (Fig. 6B–D, F). *Ephedra breana* pollen cone bracts (Fig. 6G) had similar external morphology and similar epidermis and hyaline margin anatomy to those of *E. triandra* and *E. tweediana*. The mesophyll differed in that it contained fewer (2–6) adaxial layers of cellulosic fibers and 1–2 abaxial layers of tanniniferous cells (Fig. 6H, I), and lacked mucilage cells.

Pollen cone bracts of the papery species *E. multiflora* were wide-orbicular, dry, thin, and membranous (Fig. 5G–I), with the same morphology and anatomy as seed cone bracts even at the earliest developmental stages observed (Fig. 5H, I).

### Comparative anatomy and histology of leaves

**Fig. 7:**
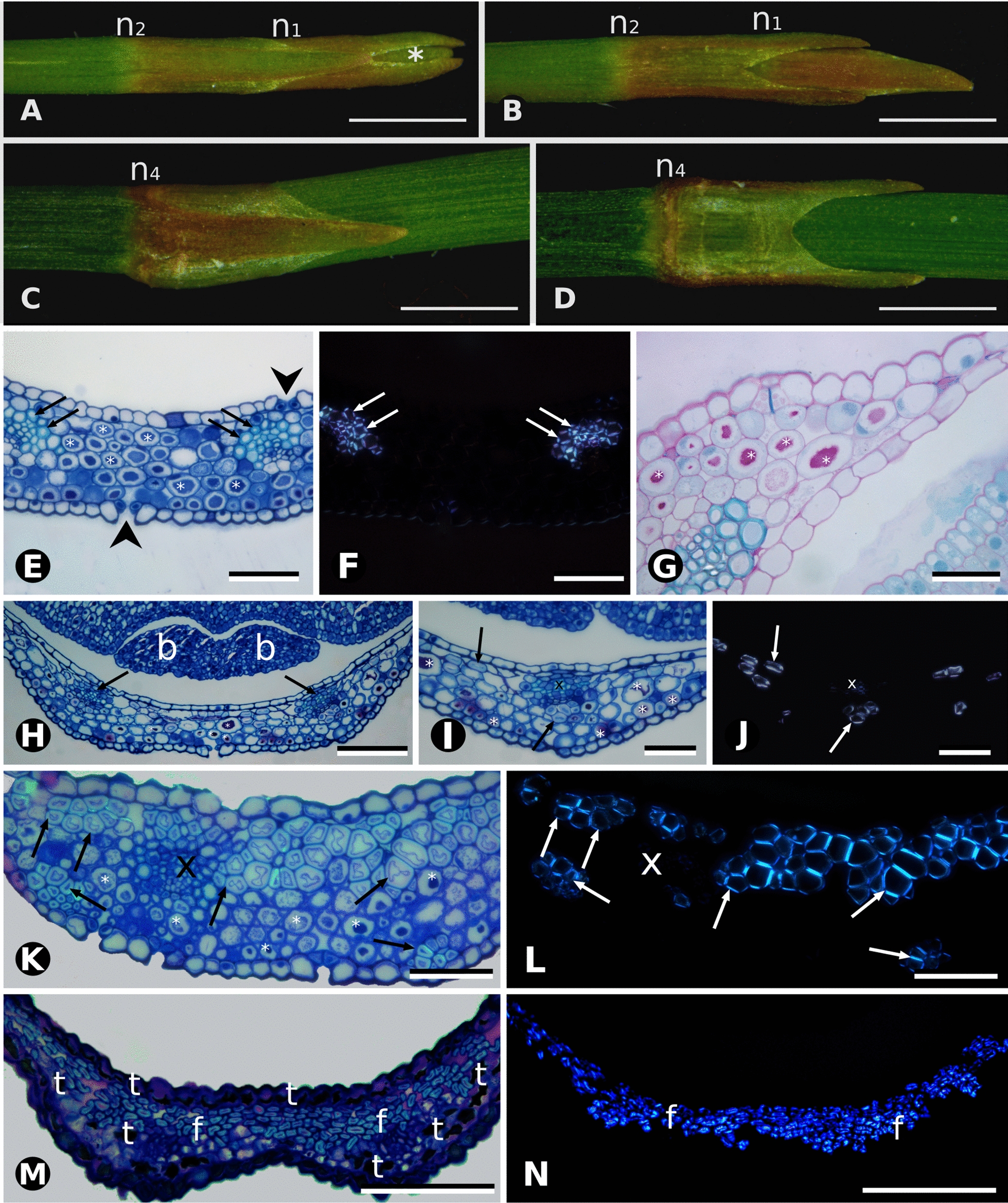
Comparative leaf morphology and anatomy of *Ephedra* in this study. **A**–**D** Overview of *E. tweediana* leaf morphology under a stereomicroscope. **A**, **B** Stem apex showing leaves from the first and second nodes in frontal and lateral views, respectively. **C**, **D** Detail of the fifth node from the stem apex showing developing leaves in frontal and lateral views, respectively. Note the development of the lateral, papyraceous zone. **E**–**G** *E. tweediana* (male individual). **E** Toluidine blue O and PAS staining. Mucilage cells (*) are present throughout the mesophyll, cellulosic fibers (arrows) are associated with vascular bundles, and stomata are present on both leaf surfaces (arrowheads). **F** Polarized light microscopy of section in **E** showing the birefringence and distribution of cellulosic fibers (arrows). **G** Positive PAS staining of mucilage in mesophyll cells. **H**–**J** *E. tweediana* (female individual). **H** General overview, arrows indicate vascular bundles, two axillary buds (b) are visible and mucilage cells (*) are distributed throughout the mesophyll. **I** High magnification of the vascular bundle in **D**, showing cellulosic fibers (arrows) associated with the bundle and also distributed throughout the mesophyll. **J** Polarized light microscopy of section in **I** showing the birefringence of xylem cells (x) and cellulosic fibers (arrows). **K** *E. triandra* (male individual), with a different pattern from that found in *E. tweediana*: cellulosic fibers (arrows) are associated with vascular bundles, and also present in bundles abaxially and in layers adaxially. Mucilage cells are found in multiple layers on the abaxial surface. **L** Polarized light microscopy of section in **K** showing the distribution and birefringence of the cellulosic fibers. **M** *E. multiflora* (female individual) with tannin cells (t) in the adaxial epidermis and also distributed in one or two layers on the abaxial face. Cellulosic fibers (arrows) differentiate in several adaxial layers in the mesophyll. **L** Polarized light microscopy of section in **K** showing the distribution and birefringence of the cellulosic fibers. Node 1 (n1), node 2 (n2), node 4 (n4). Scale bars: 1 mm (**A**–**D**), 100 µm **(E**, **F**, **K**–**N**); 50 µm (**G**, **I**, **J**); 200 µm (**H**)

Male and female individuals within a species share the same, highly reduced leaf morphology and anatomy, starting green and becoming dry and papery at maturity (Fig. 7A–D). Leaf development in *E. tweediana* can be summarized into six stages: (1) leaf primordium (apical bud) without hyaline margins and undifferentiated mesophyll; (2) leaf primordium with incipient hyaline margins (3–5 cells wide) and undifferentiated mesophyll; (3) young leaf (second node) with wider hyaline margins (7–15 cells wide), compact parenchyma and mucilage chlorenchyma; (4) compact parenchyma differentiated into cellulosic fibers; (5) mature leaf (fifth node) with hyaline margins, cellulosic fibers and tanniniferous cells; and (6) senescent leaf without tanniniferous cells. Early stages of leaf development in *E. tweediana* showed leaf primordia, with increased cell layers and cell size, and the inception and progressive growth of the hyaline epidermal margin.

Young leaves (second node) of both sexes in *E. triandra*, *E. tweediana*, and *E. breana* developed hyaline, papery margins 7–15 cells wide, comprising the overlapping adaxial and abaxial epidermis. The central longitudinal green area was relatively reduced, with a mesophyll of 1–3 adaxial layers of compact parenchyma, 5–7 abaxial layers of mucilage chlorenchyma surrounding two vascular bundles, and transfusion tissue (Fig. 7E–L). In mature leaves (fifth node), adaxial parenchyma differentiated into cellulosic fibers, and the mucilage cells found at young stages differentiated into tanniniferous cells (Fig. 7K, L). Cellulosic fibers and tanniniferous cells differentiated earlier in leaves of *E. breana* compared to *E. triandra* and *E. tweediana* (Fig. 7E–L), especially in male individuals. In *E. multiflora*, mucilage chlorenchyma was found as early as the leaf primordium stage (Fig. 5J–N, P, Q) with evidence of early transition to tanniniferous cells (Fig. 5O, R). Young leaves (second node) of *E. multiflora* developed hyaline margins 7–12 cells wide and differentiated adaxial fibers and abaxial tanniniferous cells (Fig. 7M, N), reaching mature anatomy much earlier in development.

### Histochemical analyses

Histochemical test results are reported for comparable cell types across cone bracts and leaves (Table 1; Additional file 1; Fig. 5S, T). Cutin was detected in epidermal cell walls, non-crystalline cellulose and hemicellulose in primary walls, and pectin in the primary wall and middle lamellae, as expected. Histochemical assays also revealed three unusual features: (1) presence of mucilage within chlorenchyma cells in young leaves and seed cone bracts (Stage 1) of fleshy species, in pollen cone bracts of *E. triandra* and *E. tweediana*, and in leaf primordia and early green bract stages of *E. multiflora*; (2) lack of lignin and presence of microcrystalline cellulose in the secondary wall of fibers; and (3) lack of the typical storage molecules (starch, lipids, or proteins) in bracts and leaves.

## Discussion

Here, we investigated the comparative anatomy of fleshy diaspores in South American *Ephedra*, a key aspect of their reproductive biology. We found that (1) fleshy and papery-winged seed cone bracts develop from a common early anatomy of mucilage chlorenchyma that becomes distinct at the pollination stage; (2) mucilage underlies the gelatinous texture of fleshy seed cone bracts; (3) cellulosic fibers found in bracts likely function as apoplastic water “pipes” that support bract fleshiness; and (4) seed cone bract diversification likely results from changes in the timing of leaf development, combined with a novel differentiation program repurposed from leaves. In summary, we propose that *Ephedra* bract fleshiness is a novel differentiation program repurposed from mucilage tissue present in leaves, with diversity of bract morphology and anatomy likely evolving via heterochronic processes involving changes in the timing of leaf developmental events.

### Fleshy and papery-winged seed cone bracts differentiate from shared morphology early in development

**Fig. 8:**
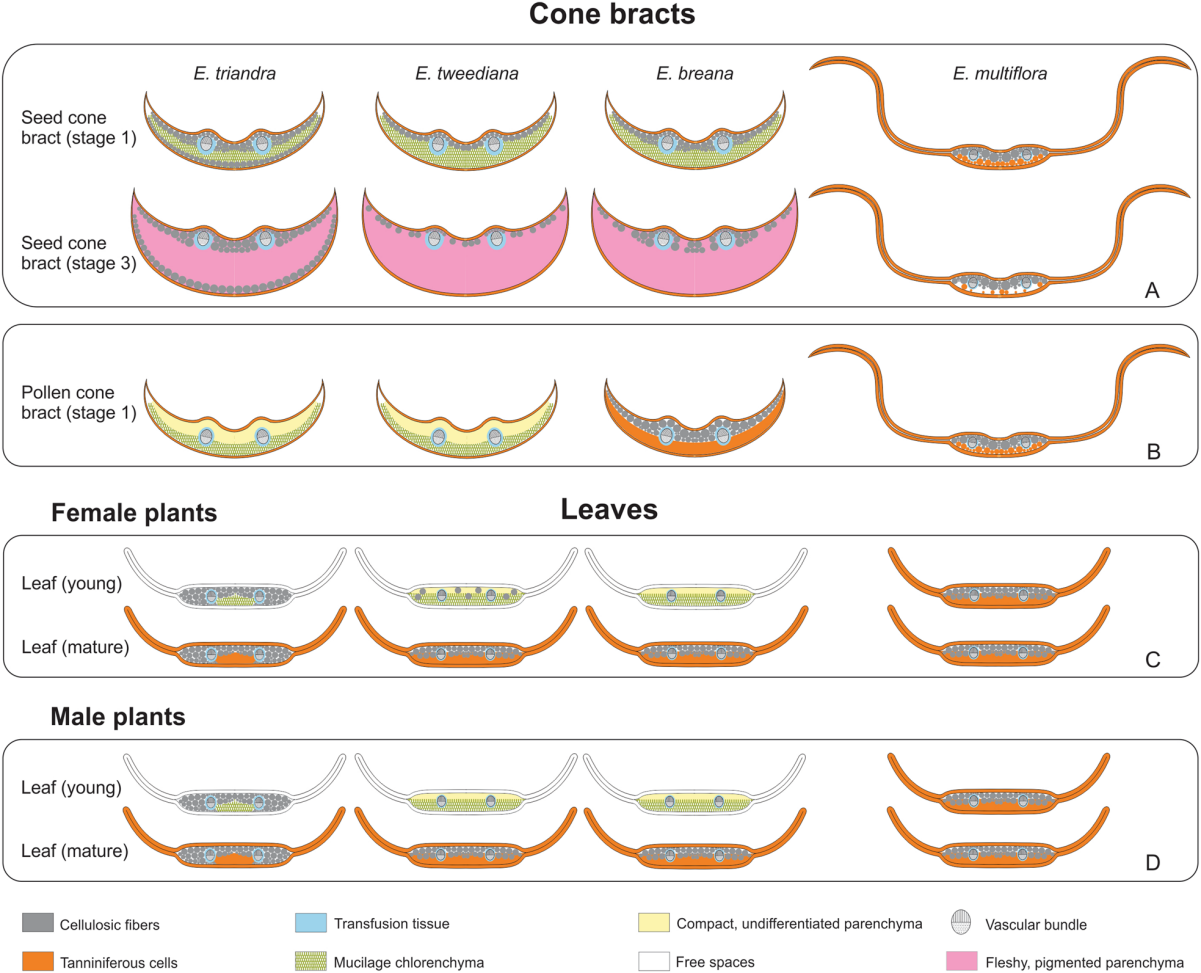
Summary diagrams of leaf and bract anatomy observations for *Ephedra breana*, *E. multiflora, E. triandra*, and *E. tweediana*. **A** Seed cone bracts at Stage 1 (first row) and 3 (second row). **B** Pollen cone bracts at Stage 1. **C** Leaves from female plants: at the second node (young, first row) and at the 5th node (mature, second row). **D** Leaves from male plants: at the second node (young, first row) and at the 5th node (mature, second row). Refer to figure inset for the meaning of colors and symbols

Our ontogenetic observations and histochemical assays provide evidence that seed cone bracts from fleshy species develop previously undescribed, highly specialized mucilage chlorenchyma discernible at Stage 1 (pollination, non-fleshy green) and Stage 2 (non-fleshy, turning from green to red). This mucilage chlorenchyma is not found in papery-winged bracts at Stage 1 (Fig. 8A), but rather in earlier green stages (Fig. 5D, S, T). Mucilage chlorenchyma is also present in pollen cone bracts of fleshy species, except for *E. breana*, where it is replaced by tanniniferous cells (Fig. 8B).

### Mucilage underlies the gelatinous texture of *Ephedra* fleshy seed cone bracts

Seed cone bracts of *E. breana*, *E. triandra* and *E. tweediana* have uniform, mucilage chlorenchyma mesophyll at the pollination stage that becomes distinctly red and fleshy at the seed dispersal stage. Our histochemical assays identified mucilage as the substance responsible for the gelatinous texture of the mature red bracts of *E. breana*, *E. triandra* and *E. tweediana* seed cones. Mucilage comprises a family of highly branched, polymerized carbohydrates including l-arabinose, d-galactose, l-rhamnose, d-xylose, and galacturonic acid in various proportions that may also contain glycoproteins and other compounds such as tannins [17]. While we found mucilage inside the cytoplasm of cells of a specialized type of parenchyma in *Ephedra*, mucilage is only found outside the cell or in specialized cells known as idioblasts in angiosperms [18–20]. Our analysis was not able to detect the type and location of the pigment/s responsible for the red color at later developmental stages. Anthocyanins have been reported in fleshy red gymnosperm bracts and seeds [21], and rhodoxanthin is another plausible candidate found in *Ephedra monosperma* leaves [22].

### Cellulosic fibers as apoplastic “water pipes” in seed cone bract fleshiness

Another novel observation was the presence of cellulosic fibers in *Ephedra* cone bracts and leaves. Cellulosic fibers are similar to G-fibers in overall morphology, both are an infrequent cell type in gymnosperms only known otherwise from the wood of certain conifers [23], roots of cycads, and vegetative organs of Gnetales [24–27]. However, they have differences in structure and function: G-fibers or gelatinous fibers (also known as tension fibers) are long, fusiform, unbranched cells with lignified primary and secondary walls and a non-lignified innermost wall or gelatinous layer (Sg, or G-layer), composed mostly of lamellate cellulose [26]. Cellulosic fibers are also long and fusiform, but they are usually branched, with non-lignified primary and secondary walls and a tertiary wall of microcrystalline cellulose. In this study, we adopt “cellulosic fibers” to distinguish a particular type of fiber without lignin from the typical G-fibers, which usually differentiate in response to external forces, such as gravity or wind [26]. The specialized cell wall structure of G-fibers allows them to contract and produce counteracting tensile forces to reorient organs [28], as in Gnetales stems and roots [25]. Cellulosic fibers are only known for Gnetales within gymnosperms: as hypodermal fibers in *Welwitschia* leaves [24, 29], as cellulose fibers in *Gnetum* leaves [25], and also misidentified as G-fibers in the cortex of *Ephedra* stems [27]. In *Gnetum* leaves, cellulosic fibers perform a supplementary apoplastic water transport function to compensate for the low density of leaf veins [30], rather than the typical mechanical function. A water transport function is also compatible with a putative role in protecting axillary buds (leaves), and reproductive strobili (pollen and seed cone bracts) against desiccation early in development, especially relevant in the semi-arid to arid environments inhabited by *Ephedra* in general [14], and the South American species in particular (Fig. 1). Even though these cellulosic fibers have an overall function as a supplementary water supply, this water has apparently been specifically deployed to generate a new structure (the fleshy bracts) in *Ephedra* seed cones to aid in seed dispersal.

### Shared leaf anatomy across species differs in the timing of maturation

Mature *Ephedra* leaves are typically reduced, scale-like, dry and membranous, and whither early, transferring photosynthetic function to the green stems [31]. These leaves consist of an epidermis collapsed to different degrees and lacking mesophyll, except in *E. altissima*, which develops linear leaves with little anatomical differentiation [31]. Our histological observations confirmed the reduced structure of leaves, while highlighting that they still contain distinct types of mesophyll cells at maturity: adaxial cellulosic fibers, abaxial tanniniferous cells, transfusion and vascular tissues (see also [15]). This mature leaf anatomy is similar between male and female individuals of the same species, and between species with fleshy and papery-winged seed cone bracts (Fig. 8C, D). Young leaves of fleshy species develop adaxial compact parenchyma and abaxial mucilage chlorenchyma prior to differentiating cellulosic fibers or tanniniferous cells, respectively. The transformation of chlorenchyma cells into tanniniferous cells is consistent with tannins being synthesized in chloroplast-derived organelles (tannosomes) [32, 33] and accumulating in the vacuole. Leaves of fleshy species differ slightly in the timing of maturation, having different degrees of cellulosic fiber differentiation at the young stage (Fig. 8C, D). Leaf development in the papery species *E. multiflora* has a noticeable difference in timing, with mucilage chlorenchyma and tanniniferous cells present much earlier, at the leaf primordium stage, and mature leaf anatomy already present in the first node. Despite being non-photosynthetic and highly reduced, mature *Ephedra* leaves are not completely dead structures, since tanniniferous cells are alive and cellulosic fibers can potentially continue to supply water via apoplastic transport. Considering all the evidence, mature leaves more likely perform a protective function towards axillary buds, against dehydration, heat, UV radiation [34] and herbivory [35].

### Repurposing and heterochrony in seed cone bract evolution and diversification

Mucilage cells found in fleshy seed cone bracts are also found in young leaves, but while they turn into tanniniferous cells in leaves, they produce red pigment and expand into fleshy structures in seed cone bracts instead, presumably by increasing water intake. We propose that the terminal “leaf differentiation program” (mucilage cells becoming tanniniferous cells) fulfills a protective function that has been repurposed into a “fleshy bract differentiation program” (mucilage cells dividing, expanding, becoming fleshy and red) with a novel, seed dispersal role. Papery-winged bracts of *E. multiflora* pollen and seed cones follow the differentiation program of leaves, as do pollen cone bracts of *E*. *breana* (Fig. 8). Our results also revealed that reproductive bracts are anatomically more diverse than vegetative leaves in *Ephedra*, not due to new cell types, but to novel combinations enabling alternative differentiation pathways that depart from baseline leaf development. When comparing and contrasting bract and leaf anatomy (Fig. 8A, B vs. C, D), the structure of pollen cone bracts can be (a) the same as seed cone bracts and leaves (*Ephedra multiflora*); (b) different from seed cone bracts but similar to leaves (*E. breana*), or (c) different from seed cone bracts and leaves (*E. tweediana* and *E. triandra*). Sexual dimorphism in cone bracts in the fleshy species’ is presumably related to their respective functions in pollen protection vs. animal seed dispersal via independent selective forces. At first sight, this reasoning appears to fail to explain why pollen and seed cone bracts of *E. multiflora* would be similar, until we consider the plant in its natural habitat: the selective pressures for dispersal by wind at high elevation desolate environments, where animals are scarce and wind speeds are high (Fig. 1G and Additional file 2).

**Fig. 9:**
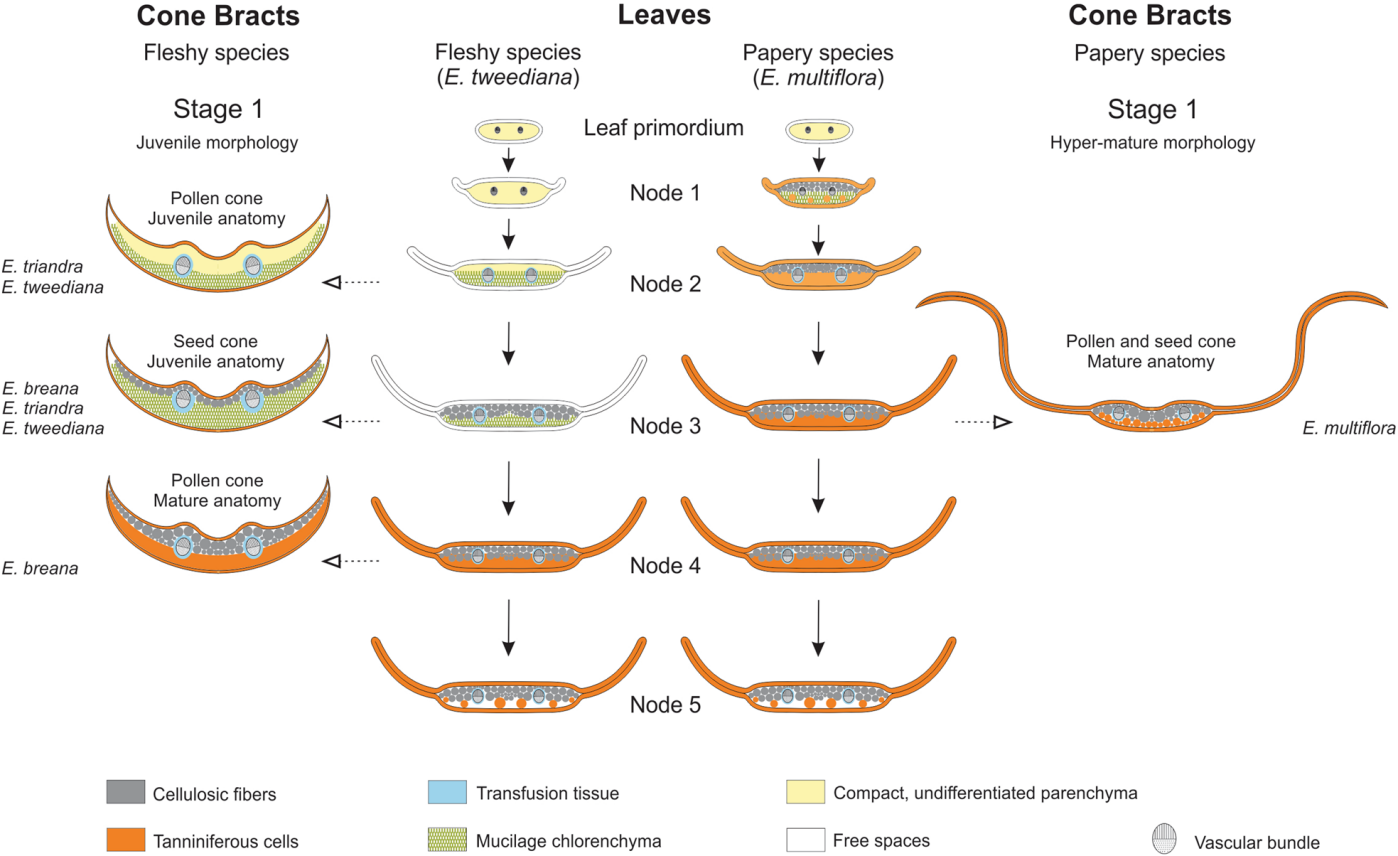
Cone bracts from leaves: Evolutionary developmental model for the repurposing of anatomical features from vegetative leaves to reproductive bracts in *Ephedra* (Gnetales). Center: Diagrams of leaf development in *E. tweediana* (left), and *E. multiflora* (right) showing the shift of mature anatomy to younger stages in *E. multiflora*. Left: Bracts of fleshy species at Stage 1, grouped by their juvenile external leaf morphology (reduced hyaline margins), showing juvenile or mature leaf anatomy depending on the species. Right: Bracts of papery species at Stage 1, with hyper-mature external leaf morphology (widely developed hyaline margins) and mature leaf anatomy. Solid-line arrows connect stages of leaf development; broken-line arrows indicate corresponding anatomies between stages of leaf development and bract structure. Fleshy species, species with fleshy seed cone bracts; Papery species, species with papery-winged seed cone bracts. Refer to figure inset for the meaning of colors and symbols

From an ontogenetic perspective, bract diversity could be explained based on changes in the timing of leaf development, and the subsequent establishment of an alternative program. On the one hand, pollen and seed cone bract morphology in fleshy species resembles juvenile leaf shape, with reduced hyaline margins (Fig. 9). The increase in cell layers of mucilage chlorenchyma in seed cone bracts as they mature can be interpreted as an extension of the juvenile leaf stage [36]. Thus, fleshy seed cone bract development would represent an alternative trajectory to normal leaf development built upon a juvenile stage. On the other hand, papery-winged bracts of *E. multiflora* reflect hyper-mature external leaf morphology (wide hyaline margins) prior to pollination stage, combined with mature mesophyll anatomy by pollination stage (Figs. 8 and 9). It could therefore be argued that papery-winged bracts emerge because the ancestral fleshy developmental trajectory has been turned off. Alternatively, and given that bracts are modified leaves, it may be more parsimonious to argue that the fleshy bract development program is never turned on in the first place, but rather reverts to a leaf program default. In brief, the evolution of fleshy bract structure may be interpreted as an alternative developmental trajectory based upon juvenile leaf stages, while that of papery-winged bracts would result from mature or hyper-mature leaf stages.

Heterochrony represents changes in the timing of developmental events leading to phenotypic variation between an ancestor and its descendants [37, 38]. Different types of heterochrony have been described: interspecific vs. intraspecific [39], growth vs. sequence [40], and transcriptional vs. metabolic vs. cellular [36]. Our proposed changes in the timing of developmental events implies a modification of homologous structures on the same individual, since leaves and bracts are considered serial (iterative) homologous structures [41], yet the adoption of heterochrony for serially homologous structures has not been fully developed [42, 43]. Fleshy seed cone bracts is an ancestral condition in the South American *Ephedra* clade [16, 44], and papery-winged bracts have evolved only once in the branch leading to *E. multiflora* and *E. boelckei* [14, 16]. On that basis, a shift in the timing of leaf maturation between *E. tweediana* (fleshy species) and *E. multiflora* (papery species) can be understood as a peramorphic (pre-displacement) change [36]. Thus, bract evolution in *Ephedra* seems to have been driven by a combination of heterochrony and an alternative trajectory during leaf development. Formal ancestral character reconstruction of anatomical and morphological traits at the genus level would provide further phylogenetic evidence for the potential role of heterochrony in cone bract evolution in *Ephedra*.

### Evolutionary considerations

Overall, bract anatomy was more nuanced than expected: remarkably similar between sister species *Ephedra tweediana* and *E. triandra* (both for pollen and seed cone bracts), yet markedly different from *E. breana* pollen cone bracts [14, 44]. Here, we contribute anatomical and histological evidence for *Ephedra* cone bracts that suggests that the ancestral fleshy character state may represent a novel type of differentiation built upon young leaf anatomy, while the derived dry, papery-winged state is likely built upon an existing differentiation pattern found in mature vegetative leaves. Additionally, we propose that cellulosic fibers observed here in *Ephedra* leaves and bracts, and also found in *Gnetum* [45] and *Welwitschia* [24, 29], may constitute a previously overlooked valuable synapomorphy for the order Gnetales.

## Conclusions

Our study revealed that fleshy and papery-winged bracts develop from a common initial anatomy shared with leaves that becomes distinct at the pollination stage. Mucilage, found in leaves and bracts at early developmental stages, provides the gelatinous texture in fleshy seed cone bracts. This mucilage appears to have been repurposed from a role in drought and freezing tolerance in leaves, to one in seed dispersal in seed cone bracts. Cellulosic fibers found in bracts and leaves likely channel extra apoplastic water, supplementing mucilage hydration during fleshy bract development. In closing, we propose that the two types of bract morphology in South American *Ephedra* arose from heterochronic changes in leaf development: fleshy bracts from the retention and repurposing of young leaf features, and papery-winged bracts from the development of hyper-mature leaf morphology. Our results further highlight the need for additional comparative anatomical studies in other fleshy gymnosperm diaspores.

## Materials and methods

### Plant materials

Reproductive branches were collected from male and female individuals of four dioecious species of *Ephedra* (Additional file 2) in natural populations in Northwest Argentina (La Rioja and Catamarca provinces) and fixed immediately in FAA (formaldehyde:alcohol:acetic acid) [46]. Species were identified following [47]. Vouchers were deposited in the herbarium at Instituto de Botánica Darwinion (SI, Buenos Aires, Additional file 3), where samples were brought for processing and study. To test whether cone bract anatomy matches that of leaves, we compared young (green) and mature (brown) leaves from male and female individuals to pollen and seed cone bracts, respectively. To consistently compare similar developmental stages, we sampled the second pair of leaves from the shoot apex for the young stage, and brown leaves from unbranched basal nodes (5th node from the shoot apex) for the mature stage. Young and mature leaves of *E. tweediana*, *E. breana*, *E. multiflora*, and *E. triandra* were fixed in FAA. Additional leaves for the four species were sampled from herbarium vouchers, rehydrated and fixed following [48]. To further investigate the full scope of leaf development, we studied earlier stages in *Ephedra tweediana* and *E. multiflora*, including leaf primordia within the bud and node 1 leaves (the outermost pair enveloping the apical bud).

### Anatomy and histology

Bract anatomy and histology was conducted in pollen cones (staminate, or male) and seed cones (ovulate, or female) of four species of *Ephedra* in an ontogenetic series. We identified three developmental stages in fleshy seed cone bracts: (1) non-fleshy green; (2) non-fleshy reddish (turning from green to red), and (3) fleshy red. Stage 1 was the longest, comprising most developmental processes, from young ovule to advanced embryo. We chose bracts at the pollination stage (when ovules are green, with turgent micropylar tubes that secrete pollination drops) as a reference point for comparison among species within Stage 1. In Stage 2 the embryo is almost at its final size, and Stage 3 corresponds to mature seeds before dispersal. Pollen cone bracts were studied at anthesis (the pollen-shedding stage, with bracts comparable to Stage 1 seed cones). In order to compare the two bract types found in South America, papery-winged seed cones of *Ephedra multiflora* were also analyzed at Stages 1 and 3. Since these bracts were already fully differentiated at Stage 1, we characterized an even earlier stage for this species. Bracts and leaves at different developmental stages were dissected, dehydrated, and embedded in Technovit 7100 historesin (Kulzer GmbH, Wehrheim, Germany). Transverse Sections 2–5 µm thick were obtained using a Jung 2055 microtome (Leica, Wetzlar, Germany) following [49].

### Histochemistry

Tissue sections were stained with toluidine blue and periodic acid Schiff reaction (PAS) for general structure analysis. PAS reaction was also used for the identification of total non-soluble polysaccharides, polarized light microscopy to identify microcrystalline cellulose, Lugol’s iodine test for starch, Coomassie blue for proteins, Sudan black for lipids, ruthenium red for pectins [46], and Alcian blue for mucilage [50]. We performed the same set of histochemical tests on bracts and leaves.

### Microscopy and photography

Digital images were taken with a Nikon FXA microscope and NIS-elements software, with enhanced contrast and white balance tools. Figures were assembled in Inkscape 1.0.

## Supplementary Information

**Additional file 1. **Histochemical analyses of the mucilaginous mesophyll of seed cones of *E. triandra*, *E tweediana* and *E. breana* (in that order throughout). **A–C** Toluidine blue O showing negative stain in the cell vacuole. **D–F** Strong PAS staining in cell vacuole denotes the presence of insoluble polysaccharides (*) in mesophyll cells. **G–I** Combined staining of toluidine blue O and PAS, intensely staining the vacuoles (*) of mesophyll cells indicates a large amount of insoluble carbohydrates. **J–L** Alcian Blue in the vacuole of mesophyll cells indicates the presence of mucilage. **M–O** Pectin assay with ruthenium red, showing that this polysaccharide is limited to the cell walls. **P–R** Detection of total proteins with Coomassie Blue, limited to the cytosolic region (arrows). Scale bar 50 µm.

**Additional file 2. **General characteristics of *Ephedra* species (Gnetales) investigated in this study. Data from [47], except for dispersal syndrome, which are inferred from morphology based on studies in other species [12].

**Additional file 3. **Voucher information. Location references: COUNTRY, Province, department (Argentina); COUNTRY, Region, Province (Chile).

## Data Availability

All data generated or analyzed during this study are included in this published article, and its additional information files.
