# Supplementary figures and images for: Heterochrony and repurposing in the evolution of gymnosperm seed dispersal units
Source: EvoDevo. 2022 Feb 16;13:7. doi: 10.1186/s13227-022-00191-8 (PMC8851845; doi:10.1186/s13227-022-00191-8)

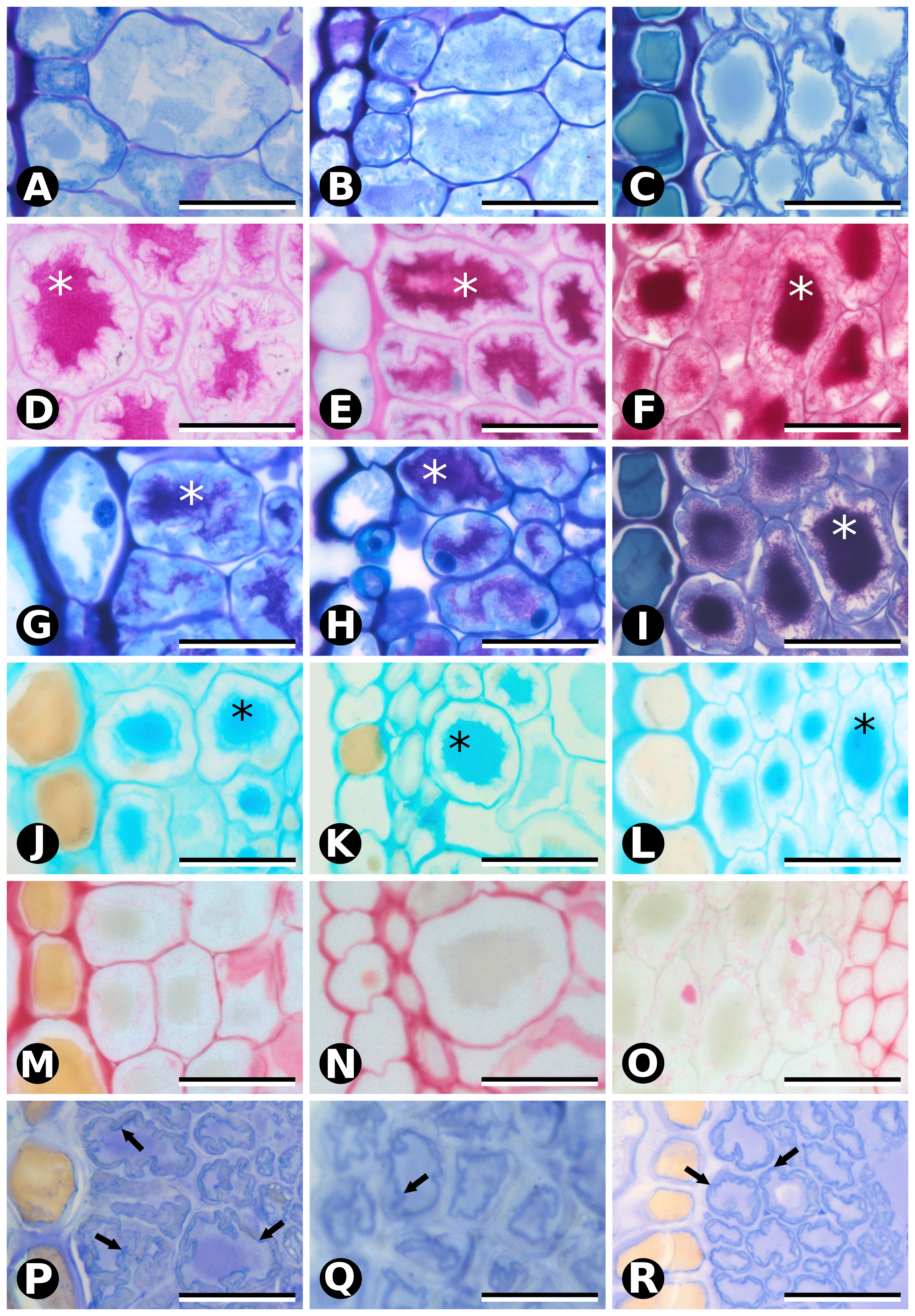

Supplement: Supplementary file 1 — Additional file 1. Histochemical analyses of the mucilaginous mesophyll of seed cones of E. triandra, E tweediana and E. breana (in that order throughout). A–C Toluidine blue O showing negative stain in the cell vacuole. D–F Strong PAS staining in cell vacuole denotes the presence of insoluble polysaccharides (*) in mesophyll cells. G–I Combined staining of toluidine blue O and PAS, intensely staining the vacuoles (*) of mesophyll cells indicates a large amount of insoluble carbohydrates. J–L Alcian Blue in the vacuole of mesophyll cells indicates the presence of mucilage. M–O Pectin assay with ruthenium red, showing that this polysaccharide is limited to the cell walls. P–R Detection of total proteins with Coomassie Blue, limited to the cytosolic region (arrows). Scale bar 50 µm. [file 13227_2022_191_MOESM1_ESM.jpg]
