# Supplementary material for: Heterochrony and repurposing in the evolution of gymnosperm seed dispersal units
Source: EvoDevo. 2022 Feb 16;13:7. doi: 10.1186/s13227-022-00191-8 (PMC8851845; doi:10.1186/s13227-022-00191-8)
Supplement: Supplementary file 2 — Additional file 2. General characteristics of Ephedra species (Gnetales) investigated in this study. Data from [47], except for dispersal syndrome, which are inferred from morphology based on studies in other species [12]. [file 13227_2022_191_MOESM2_ESM.docx]

Table S1

| **Species** | **Habitat** | **Altitude range** | **Growth habit** | **Bract Type (of seed cone)** | **Dispersal syndrome** |
| --- | --- | --- | --- | --- | --- |
| *E. triandra* | Semi-arid, on rocky or heavy soils. | 0 - 3000 | Shrub or sub-shrub | Fleshy | Birds |
| *E. tweediana* | Humid (hills and riparian woods) on heavy soils | 0 - 500 | Shrub or lianas | Fleshy | Birds |
| *E. breana* | Semi-arid, on rocky soils | 500 – 4200 | Shrub | Fleshy | Birds |
| *E. multiflora* | Dry on rocky soils | 1000 – 4000 | Shrub | Papery winged | Wind |
