# Supplementary material for: Heterochrony and repurposing in the evolution of gymnosperm seed dispersal units
Source: EvoDevo. 2022 Feb 16;13:7. doi: 10.1186/s13227-022-00191-8 (PMC8851845; doi:10.1186/s13227-022-00191-8)
Supplement: Supplementary file 3 — Additional file 3. Voucher information. Location references: COUNTRY, Province, department (Argentina); COUNTRY, Region, Province (Chile). [file 13227_2022_191_MOESM3_ESM.docx]

Table S1.

| **Species** | **Herbarium Vouchers** | **Location** | **Coordinates** |
| --- | --- | --- | --- |
| *E. breana* | SI 225832 (female)  SI 225851 (female)  SI 130922 (female)  SI 225850 (male)  SI 225831 (male)  SI 184654 (male) | ARGENTINA, **La Rioja**, Famatina  ARGENTINA, **La Rioja**, Famatina  ARGENTINA, **Neuquén**, Minas  ARGENTINA, **La Rioja**, Famatina  ARGENTINA, **La Rioja**, Famatina  ARGENTINA, **Mendoza**, Malargüe | S28°34’ W68°44’17’’  S28°56’ W67°40’  S36°49’08’’ W 71°03’10’’  S28°56’ W67°40’  S28°33’ W68°44’  S35°36’00’’ W68°40’23’’ |
| *E. multiflora* | SI 225851 (female)  SI 29237 (female)  SI 29233 (female)  SI 29139 (male) | ARGENTINA, **La Rioja**, Vinchina  ARGENTINA, **Mendonza**, San Juan  CHILE, **Antofagasta**, El Loa  CHILE, **Antofagasta**, El Loa | S28°41’ W69°10’  S32°53’37’’ W68°50’15’’  S22°18’56’’ W68°55’44’’  S22°18’56’’ W68°55’44’’ |
| *E. triandra* | SI 226176 (female)  SI 226177 (male) | ARGENTINA, **La Rioja**, Anillaco  ARGENTINA, **La Rioja**, Anillaco | S28°48’20’’ W66°56’10’’  S28°48’20’’ W66°56’10’’ |
| *E. tweediana* | SI 225834 (female)  SI 225833 (male)  SI 183874 (male) | ARGENTINA, **Catamarca**, Ambato  ARGENTINA, **Catamarca**, Ambato  ARGENTINA, **Ciudad Autónoma de Buenos Aires**, Núñez | S28°12’44’’ W65°52’33’’  S28°12’44’’ W65°52’33’’  S34°32’33’’ W58°26’33’’ |
